# Antimicrobial Activity of Grapefruit Seed Extract on Edible Mushrooms Contaminations: Efficacy in Preventing *Pseudomonas* spp. in *Pleurotus eryngii*

**DOI:** 10.3390/foods13081161

**Published:** 2024-04-11

**Authors:** Marcello Murgia, Sara Maria Pani, Adriana Sanna, Luisa Marras, Cristina Manis, Alessandro Banchiero, Valentina Coroneo

**Affiliations:** 1 Department of Medical Sciences and Public Health, University of Cagliari, Cittadella Universitaria Monserrato-S.P. Monserrato-Sestu Km 0.700, 09042 Monserrato, CA, Italycoroneo@unica.it (V.C.); 2 Analysis Laboratory, ASL Cagliari, Via Piero della Francesca, 1, 09047 Su Planu, CA, Italy; 3 Department of Life and Environmental Sciences, Blocco A, Room 13, University of Cagliari, Cittadella Universitaria Monserrato-S.P. Monserrato-Sestu Km 0.700, 09042 Monserrato, CA, Italy

**Keywords:** plant extracts, eco-friendly sustainable antimicrobials, spoilage microorganisms, edible mushrooms, yellowing

## Abstract

*Pleurotus eryngii* is an edible mushroom that suffers significant losses due to fungal contamination and bacteriosis. The Pseudomonadaceae family represents one of the most frequent etiologic agents. Grapefruit seed extract (GSE) is a plant extract that contains different bioactive components, such as naringin, and exhibits a strong antibacterial and antioxidant activity. Over the last decade, GSE use as an alternative to chemical treatments in the food sector has been tested. However, to our knowledge, its application on mushroom crops has never been investigated. This study focuses on evaluating GSE efficacy in preventing *P. eryngii* yellowing. GSE antibiotic activity, inhibitory and bactericidal concentrations, and antibiofilm activity against several microorganisms were tested with the Kirby–Bauer disk diffusion assay, the broth microdilution susceptibility test, and the Crystal violet assay, respectively. In vitro, the extract exhibited antimicrobial and antibiofilm activity against *Staphylococcus aureus* 6538 and MRSA (wild type), *Escherichia coli* ATCC 8739, and *Pseudomonas* spp. *(Pseudomonas aeruginosa* 9027, *P. fluorescens* (wild type)). GSE application in vivo, in pre- and post-sprouting stages, effectively prevented bacterial infections and subsequent degradation in the mushroom crops: none of the *P. eryngii* treated manifested bacteriosis. Our findings support the use of GSE as an eco-friendly and sustainable alternative to chemical treatments for protecting *P. eryngii* crops from bacterial contamination, consequently ensuring food safety and preventing financial losses due to spoilage. Furthermore, GSE’s potential health benefits due to its content in naringin and other bioactive components present new possibilities for its use as a nutraceutical in food fortification and supplementation.

## 1. Introduction

**Figure 1:**
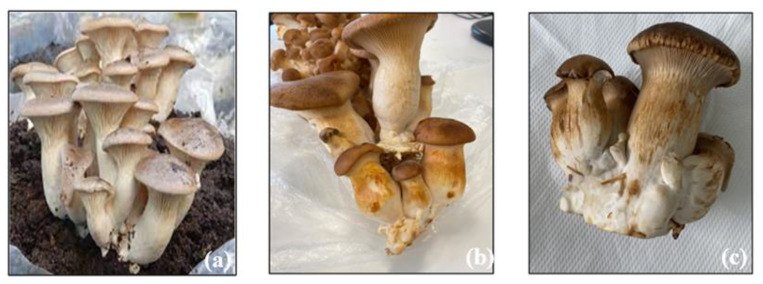
(**a**) *Pleurotus eryngii* without contamination; note the white stem without spots; (**b**) *P. eryngii* contaminated after a few days from first signs: appearance of rusty-red spots on the entire stem; (**c**) *P. eryngii* with bacteriosis after one week.

*Pleurotus eryngii* is a basidiomycete mushroom known in Italy as “cardoncello” [1,2]. It is also known as “royal trumpet” or “royal oyster” and is one of the most valuable edible mushrooms, with several varieties. Typically found in southern Europe, Northern Africa, the Middle East, and Central Asia, it is considered one of the most widely spread species of *Pleurotus* and has been known since ancient times for its excellent medicinal and nutritional characteristics [3,4,5]. This mushroom possesses a low percentage of calories and has a good concentration of major nutrients, such as protein, peptides, minerals, terpenoids, traces of various elements, fiber, and polysaccharides (*P. eryngii* polysaccharides—PEPs). These characteristics have aroused particular interest because of the anti-cancer, hepatoprotective, anti-lipidemia, immune system strengthening, and other activities shown in vitro and in animal models [6,7,8,9,10]. For this reason, *P. eryngii* is classified among functional foods [11,12,13] and used to create “healthy snacks” [14], namely, foods with high nutritional and biological value (rich in fiber and protein, low in salt, sugar, fat, and calories) [11]. Although this edible mushroom has several beneficial properties for our body, some peculiarities, such as a susceptibility to contaminations by mold and bacterial diseases [15], may compromise its quality. Generally, fungus-induced contaminations are attributed to *Cladobotryum mycophilum*, responsible for “spider’s web disease” [16,17,18], *Gliocladium roseum*, responsible for “brown spot” [19], and *Trichoderma* spp., responsible for “green mold” [20]. The bacterial blight culprits belong to several species, such as *Pantoea* spp. [14], *Erwinia beijingensis*, *Ewingella americana* [21,22], *Enterobacter amnigenus*, and *Staphylococcus* spp. However, Pseudomonadaceae, such as *Pseudomonas tolaasii* and *P. fluorescens*, are the most relevant and responsible for the stem’s yellowing [15,23,24]. It has indeed been noted that the initial colonization of the cap induces a loss in production yield; the size of the fruiting body is affected by the bacterial populations in the pre-harvest stage, consequently jeopardizing the quality of the harvested products [15,25]. The yellowing of *P. eryngii* is a bacteriosis that manifests with small yellow or light brown spots on the pileum accompanied by water-rich elongated and coalescing areas on the stem. *P. eryngii* affected by yellowing show a setback in the growth process, turn reddish-brown, and reach the state of rot (Figure 1), which manifests in the final stages with an unpleasant and nauseating odor [23,26]. The occurrence of the infection, as well as its intensity, are influenced by particular environmental conditions, such as high humidity in the growing chambers and hot muggy winds, such as sirocco [15], which are typical of the Mediterranean climate.

Innovative experiments and alternatives to the use of chemicals, potentially polluting substances, are currently being evaluated to prevent such diseases, which, with a drastic decrease in *P. eryngii* sporophores, cause considerable financial losses to producers. For example, repeated applications of white wine vinegar in fungal cultures at different concentrations have been evaluated; the acetic acid with 3% concentration has, in fact, an antimicrobial activity on *P. aeruginosa* and other bacteria [23,27]. The scientific community is focused on finding effective molecules propelling biotechnology in an eco-friendly and sustainable direction. Along this line, the present study evaluates grapefruit seed extract’s antimicrobial and antibiofilm activity on several microorganisms in vitro and in vivo, focusing on preventing *Pseudomonas* spp., in particular *P. fluorescens*., in *Pleurotus eryngii*.

Grapefruit seed extract (GSE) is a well-known plant extract with strong antibacterial and antioxidant activity [28,29]. GSE applications span from use in the food sector as a food preservative and infusion into packaging matrices [30,31] to pharmaceutics (e.g., diet supplements, wound healing, glucose and lipid blood level management, etc.) [29]. GSE contains different bioactive components, such as flavonoids, polyphenols, organic acids, and others, that are considered responsible for the antimicrobial and antioxidant activity. Different studies have investigated the mechanism of action of GSE against a wide range of microorganisms, including *Pseudomonas* spp. GSE antimicrobic activity has been attributed to the disruption of the bacterial membrane and liberation of the cytoplasmatic content [28,32,33,34]. In the literature, the difference in efficacy is reported to depend on the concentration of polyphenols, especially citrus flavonoids, such as naringin [28,35,36]. Naringin (5,7,40-trihydroxyflavanone-7-O-neohexperidoside) is a flavanone glycoside, soluble in water and metabolized by intestinal flora into its aglycone derivative, naringenin [37]. It is a molecule found in several fruits, such as grapes and tomatoes, and especially in citrus fruits, to which it attributes a characteristic bitter taste [38,39,40]. Naringin, being biologically active, expresses several beneficial proprieties in vitro and in vivo, such as anti-cancer and antioxidant activity [41,42,43]. In addition, several models show its role in decreasing the concentration of blood lipids, impacting hypertension, hyperlipidemia, and obesity conditions [39,42,44,45,46]. The possible role of naringin and naringenin as nutraceuticals against several conditions affecting human health is currently under study [47,48,49,50], opening a promising line of research relating not only to food supplements but also to food fortification.

Due to GSE’s functional properties, there has been a growing interest in using GSE in the food sector as an alternative to chemical treatments over the last decade. Several studies have investigated the efficacy and safety of GSE application on foods, whether directly, in combination with coating materials, or incorporated into edible films [29,51]. However, to our knowledge, none of these studies have evaluated the effectiveness of GSE in preventing *P. eryngii* yellowing.

## 2. Materials and Methods

From September 2022 to June 2023, specialized technical staff from the Hygiene Laboratory of Cagliari University (accredited according to UNI EN ISO IEC 17025:2017 [52]) carried out several inspections on a mushroom farm growing *P. eryngii* var. *eryngii* in the south of Sardinia (Italy). During the inspections, the technical staff evaluated mushroom contamination while sampling *P. eryngii* specimens (according to UNI EN ISO 7218:2013 [53]); eight basidiomata presenting signs of yellowing and ten without signs of yellowing were collected. Additionally, the producers assisted the technical staff in evaluating the quality of mushrooms based on their appearance, size, color, and texture. The sampled basidiomata were placed in refrigerators at 8 °C, transported to the Hygiene Laboratory of Cagliari University, and analyzed for *Pseudomonas aeruginosa*. Then, the antimicrobial and antibiofilm activity of the GSE was evaluated on *Pseudomonas* spp. and other microorganisms. The field experiment took place after in vitro testing, and GSE was atomized on the mushroom growth substrate surface before and after sprouting (see below for a detailed description of each step).

### 2.1. Grapefruit Seed Extract

The grapefruit seed extract (100 mg; DSLD (Dietary Supplement Label Database): 296039) used for this experiment was a dietary supplement produced by the certified company Solaray, est. 1973 (Park City, UT, USA) and falls under the FDA regulations for production, marketing, and sale. The company policy includes testing at three different stages during the manufacturing process (suppliers: raw materials; factory: at intake and before bottling) and up to six different quality tests of the product, including microbial testing and contaminant testing to guarantee the absence of potentially harmful chemicals and pesticides. The company facility is 455-2 GMP (Good Manufacturing Practices) certified, and its laboratory is ISO 17025:2017 [52] certified. The GSE was formulated with substances from natural origin only. The other ingredients declared by the producer were vegetable glycerin and natural grapefruit flavor, as stated on the label.

Below is a description of the methods we used to analyze the GSE to ensure the absence of synthetic compounds and measure flavonoid compounds: ultra-high performance liquid chromatography–quadrupole time-of-flight mass spectrometry analysis (UHPLC–Qtof-MS) and gas chromatography–mass spectrometry (GC–MS).

*UHPLC–Qtof-MS analysis:* To 10 μL of extract, 990 μL of methanol was added. The diluted samples were analysed with a 6560 Q-TOF/MS coupled with an Agilent 1290 Infinity II LC system (Agilent Technologies, Palo Alto, CA, USA). An aliquot of 2.0 μL from each sample was injected in a BEH Amide, 1.7 μm, 150 mm × 2.1 mm chromatographic column (Waters Corporation, Milford, MA, USA). The mobile phase consisted of water containing 0.1% formic acid (A) and a mixture of acetonitrile:methanol (9:1) with 0.1% formic acid (B), flowing at a rate of 0.150 mL/min. This phase was applied using the following linear gradient elution, starting with 85% A and 15% B for 0 min, followed by a gradual increase to 21% B over the next 8 min, then an increase to 40% B over the next 4 min, further to 60% B over the next 7 min, and finally to 90% B over the final 2 min. The mass spectrometric analysis was performed with a QToF-MS equipped with an ESI source with Jet Stream technology using the following parameters: drying gas (N2) flow rate, 11.0 L/min; drying gas temperature, 250 °C; nebulizer, 35 psig; sheath gas temperature, 325 °C; sheath gas flow, 10 L/min; capillary, 3500 V; skimmer, 65 V; Oct RF V, 800 V; fragmentor voltage, 100 V. Each sample was analysed in the mass range of *m*/*z* 100–1500. During the HPLC–Qtof-MS analysis, the standard compounds (Rutin (Sigma-Aldrich, Milan, Italy, CAS 153-18-4); Naringin (Sigma-Aldrich, Milan, Italy, CAS 10236-47-2; Hesperidin (Sigma-Aldrich, Milan, Italy, CAS 520-26-3; Naringenin (Sigma-Aldrich, Milan, Italy, CAS 67604-48-2)) were co-chromatographed with the samples under the same analytical conditions for identification purposes.

*GC–MS analysis:* To 10 μL of extract was added 90 μL of BSTFA (N,O-Bis(trimethylsilyl)trifluoroacetamide), and the mixture was placed in an oven for 15 min. After derivatization, each sample was diluted in a 1:2 ratio with hexane. A Trace 1300 gas chromatograph coupled with a TSQ 9000 triple quadrupol mass spectrometer (Thermo Fisher Scientific Inc., Waltham, MA, USA) was used for the sample analysis. The volume injection was of 1 µL in the splitless mode. The injector temperature was set at 200 °C. The gas flow rate was 1 mL/min. The column was a DB5-MS (0.25 μm, 30 m × 0.25 mm) (J&W scientific, Folsom, CA, USA). Initially, the oven temperature was set at 50 °C and held for 10 min. Then, it was increased to 300 at 10 °C/min and held at 300 °C for 10 min. Ions were recorded at 1.6 scan/s in the mass range *m*/*z* 50–550. Confirmation of sample components was performed by (a) comparison of their relative retention times and mass fragmentation with those of pure standards and (b) computer matching against NIST, as well as retention indices as calculated according to Kovats for C7–C40 n-alkane standard mixtures in dichloromethane (Sigma-Aldrich, Milan, Italy; product ID: 49452-U, Lot. LRAC3116).

*GSE preparation for analysis:* The commercial GSE had a density of 0.0033 g/mL (3333.33 μg/mL). GSE was diluted with TBS (tryptic soy broth), and different concentrations were evaluated empirically during the preparation of the laboratory tests. The target concentration identified was 52,000 μg/mL.

### 2.2. Culture Investigations

The presence and concentration of *Pseudomonas aeruginosa* were measured (UNI EN ISO 16266:2006 [54]) in the samples (*n* = 8) presenting symptoms of yellowing disease and in the samples (*n* = 10) without yellowing. The strains ATCC *P. aeruginosa* ATCC 9027 and *P. fluorescens* (wild type) were used as reference microorganisms.

### 2.3. Antimicrobial Activity—Preliminary Assay

The Kirby–Bauer disk diffusion assay was used as a preliminary assay for evaluating the antimicrobial activity of grapefruit seed extract against several microorganisms: *P. aeruginosa* ATCC 9027, *P. fluorescens* (wild type), *S. aureus* ATCC 6538, *S. aureus* MRSA wild type, and *C. albicans* ATCC 2091. In the case of *Pseudomonas* spp., the microbial suspension was prepared with *P. fluorescens* and *P. aeruginosa* previously isolated from the specimens with bacterial disease. Each suspension presented a corresponding concentration of 1 McF (OD600). In addition, Muller–Hinton medium (agar 17.0 g/L, beef infusion solids 2.0 g/L, casein hydrolysate 17.5 g/L, starch 1.5 g/L) was used in standard-diameter Petri dishes (90 mm), with a medium thickness of 4–5 mm. Then, a sterile swab dipped into the suspension was used for surface seeding, repeating this operation four times by rotating the plate 90 degrees each time. After the inoculum absorption, three 6 mm paper discs impregnated with the grapefruit seed extract were positioned on the growth medium. The plates were incubated at 37 °C for 24 h ± 2. Readings were taken the following day by measuring the diameter (mm) of the inhibition halos and obtaining values comparable to standard values per microbial strain, indicating them as sensitive, intermediate, or resistant.

### 2.4. Inhibitory and Bactericidal Concentration–Broth Microdilution Susceptibility Test

*P. aeruginosa* ATCC 9027 and *P. fluorescens* (wild type) inocula were placed in 96-well microplates with a concentration of 100,000 CFU/mL in nutrient broth with the test substance at different concentrations. A set of positive and negative controls was run in triplicate with the samples to ensure reliability. Negative controls consisted of culture broth and GSE to test sterility; positive controls were placed in the microwells with the culture broth and without any treatment to verify inoculum vitality. The same technique was used for *S. aureus* ATCC 6538, *S. aureus* MRSA wild type, *Escherichia coli* 25922, and *C. albicans* ATCC 2091. The MIC (minimum inhibitory concentration) and MBC (minimum bactericidal concentration) were determined [55]. After 24 h of incubation, the results were read by observing the formation of a pellet; MIC was read (first microwell with no growth), and MBC was determined (first well with no growth after the transfer of a given volume into universal agarized medium).

### 2.5. Antibiofilm Activity

A crystal violet assay [56] was applied to evaluate the antibiofilm activity of grapefruit seed extract on the microorganisms targeted. The microbial strains were revitalized in TSB (tryptic soy broth) for 24 h at 37 °C. Then, 100 µL of the microbial suspension, equal to 0.5 McF with OD600, was transferred to three wells of the microplate with supplementation of 10 µL of 1% glucose solution and incubated at 37 °C for 24 h without agitation. Positive and negative controls and the samples were run in triplicate. For positive controls, 100 μL of microbial suspension was prepared by adding 10 µL of the glucosate solution and 100 µL of 1% DMSO. For negative controls, 100 µL of TBS, 100 µL of grapefruit seed extract at the target concentration, and 10 µL of 1% gluconate solution were added. In the next 24 h of incubation, TBS was withdrawn with a micropipette and replaced with TBS-containing grapefruit seed extract at the target concentration. The controls did not undergo any treatment. After 24 h of incubation at 37 °C, the suspended medium was removed from the treated and positive controls in all microwells, and the formed biofilms were subjected to three washes with 300 μL of 0.01 mol phosphate-buffered saline (PBS, pH = 7.4) to remove weakly bound cells. It was then allowed to dry for one hour inside a thermostat at 37 °C. In the second step, the cells, bound on the surface, were fixed with 200 μL of methanol for 20 min. Excess methanol was removed and allowed to dry for 24 h. Next, staining with 200 μL of 2% Hucker’s Crystal Violet for 15 min was performed. The biofilm thus impregnated was washed three times with 300 μL sterile deionized water to remove the unbound dye and then dried at room temperature for 30 min. Then, 200 μL of the biofilm-bound crystal violet was dissolved in 33% glacial acetic acid, and absorbance was measured at 570 nm (Cary 60 UV–Vis Spectrophotometer). The percentage of biofilm eradication was calculated using the following formula (*O.D.*: optical density):

$$\%\;of\;eredication=\frac{O.D.\;positive\;control-O.D.\;treated}{O.D.\;positive\;control}\times 100$$

### 2.6. Field Experiment

The field experiment was conducted at the mushroom farm from September–October 2023. The external climatic conditions were characterized by temperatures ranging from 19 to 29 °C in September and 16 to 27 °C in October; the average humidity was 67% in September and 72% in October. An optimal microenvironment for the growth of cardoncello was guaranteed by a breathable cloth cover and a wooden support one meter above the ground on which the pre-inoculated mushroom substrate blocks were placed. In addition, to prevent fungal diseases caused by vectors, netting and several traps were placed on the upper arch. The extract was sprayed on mushroom specimens treated (n = 50) at a concentration of 52,000 μg/mL; a control group was sprayed with sterile water (n = 90). The treatment lasted about 20 days for each mushroom block from before the sprouting phase to harvesting. A total of 100 mL of extract diluted in sterile saline solution was sprayed (distance between 20 and 30 cm) on the soil and treated mushrooms (about 5 mL per day diluted in 15 mL of sterile saline solution). The culture was monitored from the early stages of development to the adult stage, recording the number of specimens with bacterial disease both in treated and control groups. The spent substrate from mushroom cultivation was disposed of after the harvesting cycle.

## 3. Results

### 3.1. Preliminary Inspections

Among the basidiomata sampled during the inspections on the mushroom farm that took place from September 2022 to June 2023, *Pseudomonas aeruginosa* was found only in the ones affected by yellowing. The features of the healthy mushrooms defined by the producers during routine screenings, namely, appearance, size, and texture, were displayed to the technical staff and considered the standard for the final evaluation of treated *P. eryngii*.

### 3.2. GSE Analysis

The flavonoid compounds measured in the methanolic phase of GSE through UHPLC–Qtof-MS analysis are reported in Table 1.

The polar metabolites detected in the GSE by GC–MS analysis are shown in Table 2. No synthetic compounds were detected. The Kovats indexes calculated for the different compounds are reported in Table 2 in comparison with the Kovats indexes reported in the NIST (National Institute of Standards and Technology) database.

For HPLC–Qtof-MS and GC–MS chromatograms of GSE see Supplementary Figures S1 and S2.

### 3.3. In Vitro Analysis

**Figure 2:**
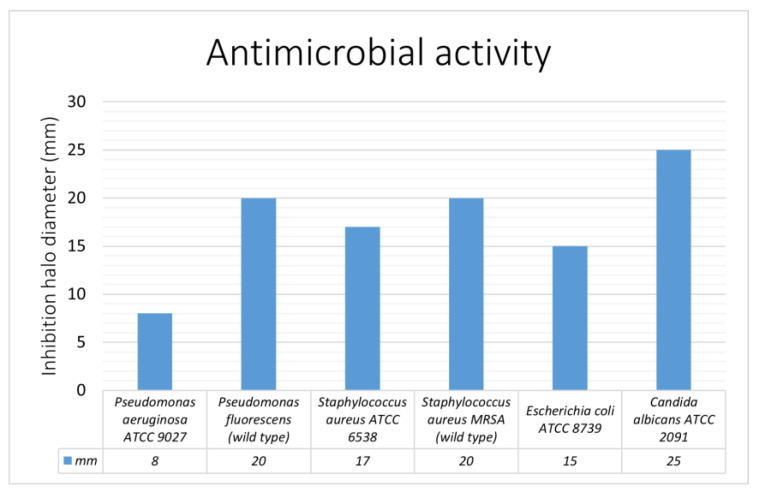
Antimicrobial activity-inhibition halo histograms. Diameters of inhibition halos (Kirby–Bauer disk diffusion assay) related to the grapefruit seed extract action are expressed in mm.

The Kirby–Bauer disk diffusion assay demonstrated the antimicrobial activity of GSE against *P. aeruginosa* ATCC 9027, *P. fluorescens* wild type, *S. aureus* ATCC 6538, *S. aureus* MRSA wild type, *E. coli* ATCC 8739, and *C. albicans* ATCC 2091. The inhibition halos for *P. aeruginosa* ATCC 9027 and *P. fluorescens* wild type were 8 mm and 22 mm, respectively. The inhibition halo diameters for each of the microorganisms tested are shown in Figure 2. 

The MIC and MBC values established through the broth microdilution susceptibility test for *P. aeruginosa* ATCC 9027, *P. fluorescens* wild type, *S. aureus* ATCC 6538, *S. aureus* MRSA (wild type), and *E. coli* ATCC 8739 are shown in Table 3.

**Figure 3:**
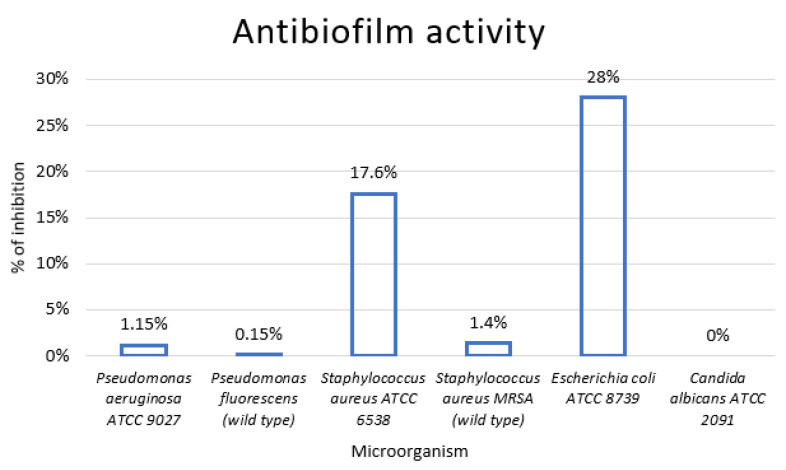
Antibiofilm activity—% of inhibition histograms. Percentages obtained with the crystal violet assay.

The percentages of inhibition established through the crystal violet assay for *P. aeruginosa* ATCC 9027 and *P. fluorescens* wild type were 1.15% and 0.15%, respectively. The percentages of inhibition for each of the microorganisms tested are shown in Figure 3.

There was no evidence of bacteriostatic and antibiofilm activity on *C. albicans* ATCC 2091.

### 3.4. In Vivo Analysis

During the field experiment, none of the *P. eryngii* treated with the grapefruit seed extract manifested bacteriosis, while three cases were observed in the controls. Field testing of the substance demonstrated protective efficacy in preventing contamination and subsequent bacterial debasement. Furthermore, according to manufacturers, the treated mushrooms were of the same quality as the healthy untreated ones, presenting the same appearance, size, color, and a slightly softer stem.

## 4. Discussion

The bacteriosis of cardoncello manifests as the appearance of reddish-brown cankers extending from the cap to the stem, inducing a change in the color and organoleptic characteristics of the product [23,57,58]. The Pseudomonadaceae family has been identified as a major culprit in the etiology of such bacterial diseases [58,59], particularly the species *aeruginosa*, pathogenic to humans, and *fluorescens*, which may cause acute opportunistic clinical manifestations of bacteremia in individuals with compromised immune systems [60]. Bacterial contamination by Pseudomonadaceae may both directly and indirectly harm the consumer since the presence of lesions on the fungus’ surface promotes contamination by other species of microorganisms [61,62]. Furthermore, a decrease in the number and quality of the fungi grown represents an important risk of economic loss for producers. In this context, our study is the first to evaluate the efficacy of the GSE against *P. eryngii* bacterial blight and to suggest its possible use for preventive purposes. Nowadays, great efforts are being devoted to finding innovative and natural alternatives to chemicals to prevent alterations in food products and guarantee food safety and maximum productivity. Pure GSE is among the several bioactive compounds originating from natural sources, and it is widely accepted and recognized as safe for direct or indirect use in food. GSE in its pure form is non-toxic and “chemical-free” (marketing term), namely, safe and environmentally friendly, containing natural ingredients only. Therefore, the use of pure GSE on food or food matrices is not expected to harm consumers and the environment. However, some commercial GSEs contain synthetic compounds [63,64], such as benzethonium chloride and benzalkonium chloride, that may derive from the conversion of unstable polyphenols during GSE extraction and purification. These compounds exhibit potent antimicrobial activity and some toxicity at high concentrations [51]. For these reasons, in the present study, we used a GSE free from these synthetic compounds. This approach enabled a reliable evaluation of GSE efficacy in protecting *P. eryngii* crops from bacterial contamination while considering safety aspects and potential environmental impacts. Therefore, we can state that the antimicrobial activity exhibited by the GSE used in this study is attributable to its natural content in polyphenols, especially flavonoids such as naringin.

The Kirby–Bauer disk diffusion assay demonstrated that the investigated GSE was active against all Gram-positive and Gram-negative bacteria, as well as *C. albicans*. GSE exhibited the largest zones of inhibition for *C. albicans*, *P. fluorescens*, and *Staphylococcus MRSA*. The crystal violet assay showed that GSE exerted antibiofilm activity on all the microorganisms tested except for *C. albicans.* In terms of percentage of inhibition, a certain variability was observed, reflecting, in our opinion, the complexity of the biofilm simulated in vitro. These findings are consistent with the literature concerning *P. aeruginosa* spp., *S. aureus* MRSA, and *E. coli* [29,32,65,66,67]. In the literature, the mechanism of GSE antimicrobial activity has been attributed to the disruption of the bacterial membrane and liberation of the cytoplasmatic content [33]. The antibiofilm effect of GSE on *S. aureus* and *E. coli* has been attributed to changes in the exopolysaccharide production rate and mobility, as well as changes in hydrophobicity in *E. coli* only [68].

The field experiment demonstrated that spraying GSE twice a day from before the sprouting phase to harvesting can prevent the growth of *P. fluorescens* and *P. aeruginosa* during the cultivation of cardoncello, which is particularly critical under several environmental circumstances. The extract antimicrobial effects were not affected by the 20–30 cm distance required for the application, suggesting that GSE is suitable as a spray. The application of GSE as a measure of prevention of bacterial blight occurrence is worth further investigation not only on *P. eryngii* but also on other foodstuffs. Furthermore, given the current attention to the nutraceutical use of naringin and other flavonoids, the potential added value of foods supplemented with GSE deserves consideration.

### Limitations and Future Directions

Since this work is the first to evaluate the effectiveness of GSE in preventing *P. eryngii* yellowing, it should be considered a pilot study. It has several limitations, such as the small number of basidiomata we were able to treat due to economic constraints of the producers. We did not conduct a challenge test with *Pseudomonas* spp. and analyzed the quality of treated mushrooms in terms of appearance, shape, color, and texture only.

The next steps to validate this natural control strategy involve (i) larger scale experimentation with at least three to five biological replicates (including 25–50 mushrooms each), which is also essential to confirm further that the slight changes in the texture due to reiterate nebulization do not affect the final quality of the products; (ii) deep evaluation of treated mushroom quality, including polyphenol oxidase (PPO) activity, sensory analysis, and chemical characterization; and (iii) conducting a challenge test with *Pseudomonas* spp. Furthermore, in the following stages, we plan to compare GSE with chemical compounds and study its mechanism of action on *Pseudomonas* spp. to gain a deeper understanding of GSE characteristics and levels of effectiveness. Moreover, considering that naringin’s antioxidant activity is affected by light and high temperatures (>100 °C) [69,70], we plan to investigate the naringin concentration and bioavailability in the final product treated with GSE.

## 5. Conclusions

*P. eryngii* yellowing is a disease that can occur in all the basiodiomata development phases, from sprouting to commercial maturation. It can bring huge economic damage to producers due to its rapid spread in *P. eryngii* cultivations and the current lack of standardized control measures. The present work contributes to the knowledge of the antimicrobial efficacy of natural GSE and provides valuable input to the branch of research aimed at preventing and controlling *P. eryngii* yellowing. Our findings support the use of GSE to protect *P. eryngii* crops from bacterial contamination, particularly from *Pseudomonas* spp., which have often been identified as responsible for the yellowing. Atomizing GSE pre- and post-sprouting represents a promising eco-friendly and sustainable control strategy alternative to chemical treatments to ensure food safety and prevent financial losses due to *P. eryngii* spoilage. In addition, due to its content in naringin and other bioactive components, GSE opens new horizons regarding its use as a nutraceutical in food fortification and supplementation. Due to the limitations mentioned above, our preliminary findings, although encouraging, require larger and deeper studies to be further validated.

## Figures and Tables

**Table 1:** Quantitative analysis and accurate mass for flavonoids determined using UHPLC–Qtof-MS. RT retention time; *m*/*z* ratio of mass to charge; Δ (ppm) mass error of an assignment when comparing a theoretical *m*/*z* and the experimentally observed *m*/*z*.

Flavonoids	RT (min)	Formula	*m*/*z Experimental*	*m*/*z Theoretical*	Δ (ppm)	Major Fragmentaion	mg/L
Rutin	6.49	C_27_H_30_O_16_	611.1606	611.1607	−0.16	303.0496	99.03
Naringin	9.75	C_27_H_32_O_14_	581.1864	581.1865	−0.17	273.0752	46.57
Hesperidin	9.25	C_28_H_34_O_15_	611.1967	611.1970	−0.49	303.0857	45.76
Neohesperidin	16.11	C_28_H_34_O_15_	611.1972	611.1970	0.33	303.0874	168.29
Naringenin	19.23	C_15_H_12_O_5_	273.0758	273.0757	0.36	287.0904	3515.05

**Table 2:** Percentage composition of polar metabolites detected in the GSE by GC–MS analysis. RT retention time. NA, not available.

RT	Compounds	%	Calculated Kováts Retention Indexes	Theoretical Kováts Retention Indexes
18.944	Lactic Acid, 2TMS derivative	14.69208	1055	1057
21.066	Diacetin, TMS	3.489502	1105	NA
22.808	Glycerol, 3TMS derivative	56.27466	1279	1282
26.651	Diacetin, TMS	5.599487	1105	NA
26.952	1,2,3-Butanetriol-3TMS	10.27547	1285	1286
27.853	Butane, 1,2,3-tris(trimethylsiloxy)-TMS	0.131453	1285	1285
28.013	Monocaproin, 2TMS	0.13052	1886	1886
28.613	Diglycerol, 4TMS derivative	0.3968	1902	NA
30.696	Ascorbic acid, 4TMS derivative	5.920048	1968	1971
31.777	9-Octadecenenitrile	0.23135	2315	NA
33.338	Citric acid, 4TMS derivative	1.752973	2618	2622
34.64	Oleamide, TMS derivative	1.105656	2763	2765

**Table 3:** Minimum inhibitory concentration (MIC) and minimum bactericidal concentration (MBC) values established through the broth microdilution susceptibility test for each of the microorganisms tested.

Target Microorganisms	MIC	MBC
*Staphylococcus aureus ATCC 6538*	162,5 µg/mL	650 µg/mL
*Staphylococcus aureus MRSA wild type*	325 µg/mL	325 µg/mL
*Pseudomonas aeruginosa ATCC 9027* *Pseudomonas fluorescens wild type*	650 µg/mL	1300 µg/mL
*Escherichia coli 8739*	650 µg/mL	1300 µg/mL
*Candida albicans ATCC 2091*	-	-

## Data Availability

The original contributions presented in the study are included in the article, further inquiries can be directed to the corresponding author.

## Supplementary Materials

The following supporting information can be downloaded at: https://www.mdpi.com/article/10.3390/foods13081161/s1. Figure S1: HPLC-Qtof/MS chromatogram of GSE; Figure S2: GC-MS chromatogram of GSE.
