# Supplementary material for: Antimicrobial Activity of Grapefruit Seed Extract on Edible Mushrooms Contaminations: Efficacy in Preventing Pseudomonas spp. in Pleurotus eryngii
Source: Foods. 2024 Apr 11;13(8):1161. doi: 10.3390/foods13081161 (PMC11049546; doi:10.3390/foods13081161)
Supplement: Supplementary file 1 [file foods-13-01161-s001.zip › foods-2908433-supplementary.pdf]

foods

Supplemental Materials

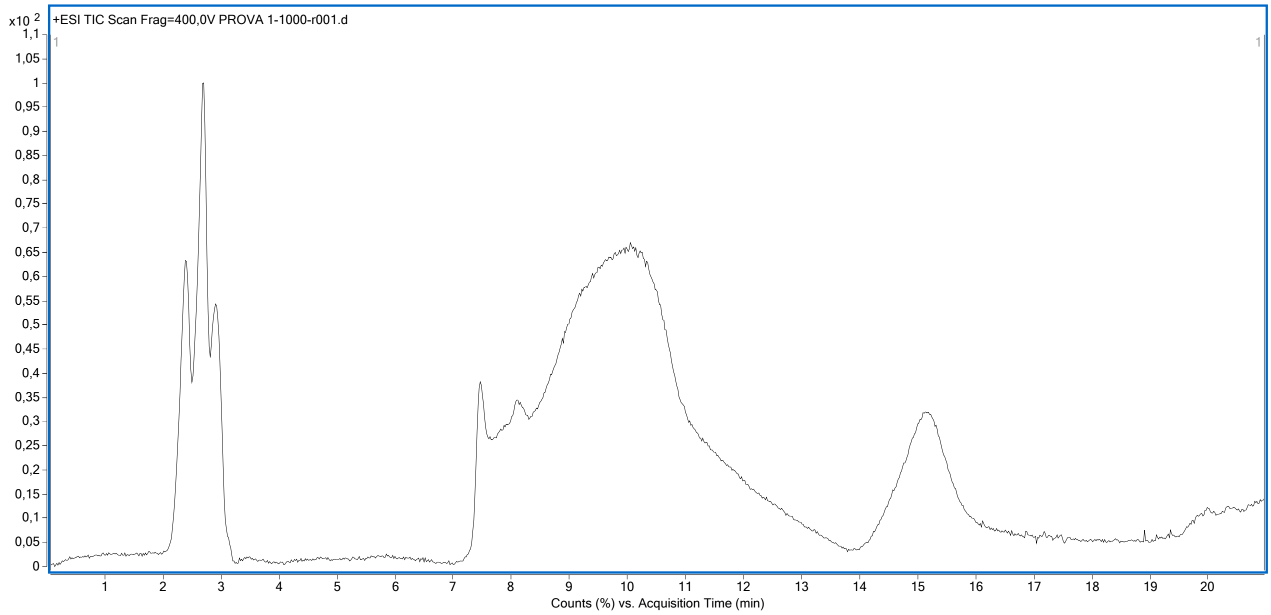

Supplementary Figure S1. HPLC-Qtof/MS chromatogram of GSE.

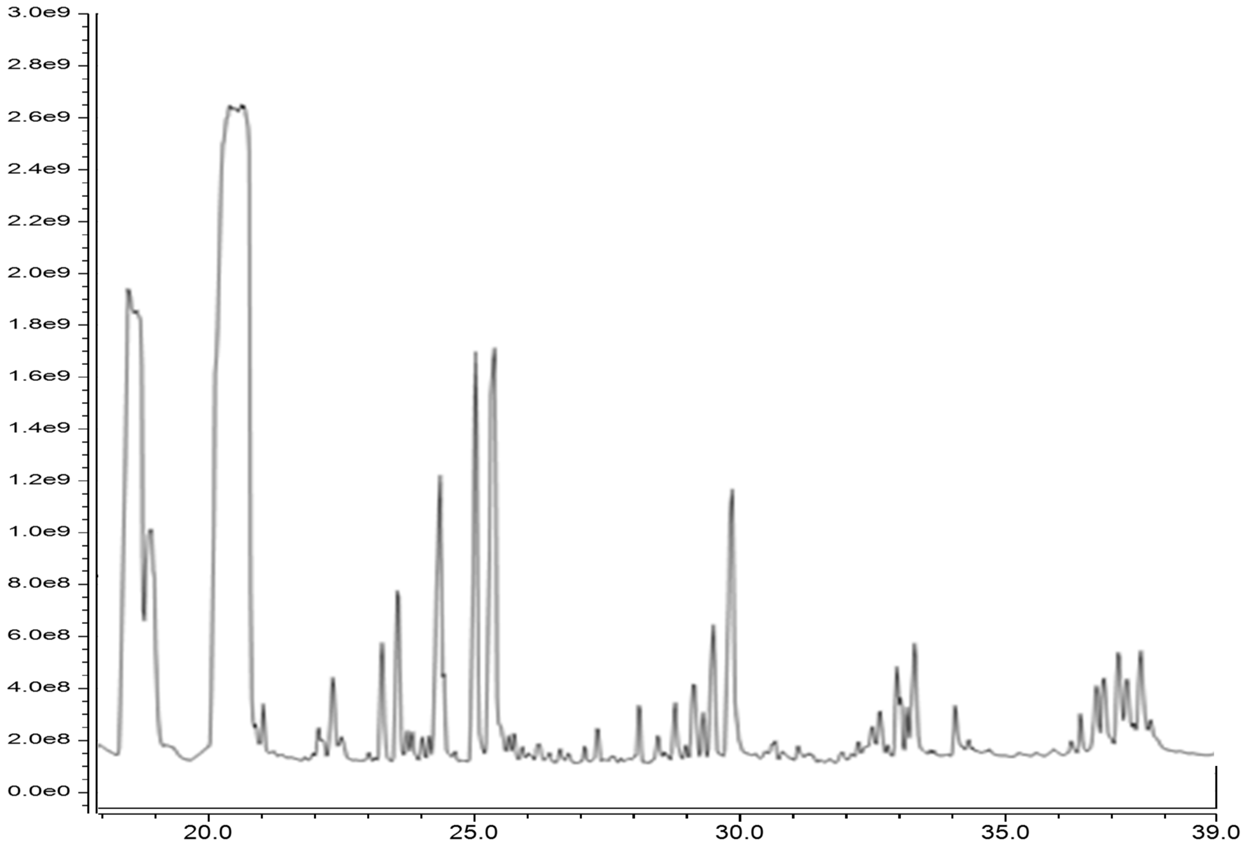

Supplementary Figure S2. GC-MS chromatogram of GSE.
